# Experience with the Implementation of Continuous Medical Education among Mother-and-Child Healthcare Providers in Ukraine: A Case Study Based on Two International Collaboration Initiatives

**DOI:** 10.3390/healthcare11131964

**Published:** 2023-07-07

**Authors:** Tetiana Chernysh, Lucas Opitz, Nataliia Riabtseva, Martin Raab, Milena Pavlova

**Affiliations:** 1 School of Health Care Management, National University of Kyiv-Mohyla Academy, Skovorody Street 2, 04655 Kyiv, Ukraine; 2 Ukrainian-Swiss Project “Medical Education Development” Implemented by the Swiss Tropical and Public Health Institute, Switzerland, Liuteranska Street 6-B, 01001 Kyiv, Ukraine; 3 Neonatal Intensive Care Unit—NICU, Pôle d’Anesthésie Réanimation, Teaching Hospital Archet 2, Le Centre Hospitalier Universitaire—CHU de Nice, 151 rte St Antoine, 06200 Nice, France; lucas.opitz@orange.fr; 4 Ukrainian Healthcare Centre, 45 Vozdvyzhenska Str., 04071 Kyiv, Ukraine; natalia.riabtseva@gmail.com; 5 Swiss Centre for International Health, Swiss Tropical and Public Health Institute, Kreuzstrasse 2, 4123 Allschwil, Switzerland; martin.raab@swisstph.ch; 6 Department of Health Services Research, Care and Public Health Research Institute—CAPHRI, Maastricht University Medical Center, Faculty of Health, Medicine and Life Sciences, Maastricht University, P.O. Box 616, 6200 MD Maastricht, The Netherlands

**Keywords:** continuous medical education, mother-and-child health, primary healthcare, simulation center, evidence-based medical practice, infrastructure development, Ukraine

## Abstract

Background: Healthcare labor market shortages due to migration, inadequate investments, and lack of continuous training are essential concerns in the Eastern European region. This article aims to describe and reflect on the experience with the implementation of continuous medical education among mother-and-child healthcare providers in Ukraine, including achievements, challenges, and barriers. We analyze this case based on two international collaboration initiatives: the Swiss–Ukrainian program in mother-and-child health that ran from 2000 to 2015, supplemented by the recent Ukrainian–Swiss project “Medical education development” in 2018–2023. Methods: We use a case study approach as the methodology for our study. We collected data from documents (project reports reviews) and in-depth interviews with stakeholders. We apply the method of directed qualitative content analysis. Results: As a result of the Swiss–Ukrainian collaborations, the knowledge and awareness of medical personnel were greatly improved. Modern clinical concepts not well understood at the outset became commonplace and were incorporated into clinical activities. Nevertheless, obstacles to the implementation and rapid uptake of changes were found in the lack of knowledge of the English language among medical doctors, the fear of changes, and the lack of openness and readiness for novel evidence-based clinical practices. However, primary healthcare practitioners in this new project seem to be more inclined to change. Conclusions: A modernized continuous medical education which is based on the values of openness, respect, dialogue, and professionalism can be implemented with the input of an international assistance program despite the resistance of the system towards change.

## 1. Introduction

Healthcare labor markets are experiencing growing shortages in terms of an insufficient number of healthcare professionals and a lack of specific skills to tackle emerging health and environmental risks. The increased use of technology is not sufficient to fill the healthcare labor gap. This creates a mismatch between supply and demand in healthcare. The problem has manifested itself in all geographical regions, though to a different extent within and between countries [1]. There is a need to revisit current education programs and ensure the necessary competencies of healthcare professionals for tackling the increasing burden of diseases, especially the combined burden of communicable and non-communicable diseases. In addition, more healthcare professionals need to be trained, which can be facilitated by investing in education infrastructure, improving entry procedures to basic medical and health education programs, and providing incentives through public policy for participation in life-long learning programs [1,2].

Such labor market challenges have been reported for the maternal healthcare sector as well. The shortage of skilled maternal healthcare providers results in overburdened and fatigued staff, which affects their well-being as well as the quality of services they offer [3]. Insufficient staff at maternal healthcare facilities means less time for contact with the women during their prenatal or post-natal visits and reduced access to care. In addition, a lack of adequate training in patient communication, support, and compassion has been linked to poor service provision [4]. Notably, maternal healthcare providers who have not received specialized communication training are not always able to recognize the needs of their clients, and their attitudes are sometimes (perceived as) disrespectful toward expectant mothers. Modern training approaches are needed to improve the experience with maternal healthcare provision for both women and service providers. Funding for scaling up innovative study programs is essential to ensuring sustainable diffusion of good practices across countries and settings [3].

In the Eastern European region, the shortages in the healthcare labor markets described above, including insufficient staff and lack of skills in the maternal healthcare sector, are even more pronounced than in other parts of Europe [5]. As in Western Europe, the aging workforce is a challenge in Eastern Europe. However, one region-specific factor intensively discussed in the literature is the high mobility of healthcare professionals to Western European countries with more attractive working conditions. Inadequate and insufficient healthcare investment due to budgetary constraints and lower priority given to healthcare issues can help to explain the large healthcare labor gap in the Eastern European region [2]. As indicated by the World Health Organization (WHO), geographical areas in Europe with lower healthcare workforce density tend to underperform in terms of maternal mortality and other health indicators [6]. Modernized education programs to increase the quantity and quality of healthcare professionals in Eastern Europe, including those in the maternal healthcare sector, are essential for achieving better health outcomes and improving experiences of care.

This article focuses on modernized approaches to continuous medical education (CME) for medical personnel of maternity wards and hospitals in Ukraine. In this paper, continuous medical education refers to the life-long professional education of medical personnel who have completed post-graduate schools; it covers a variety of activities, including different types of training, simulation courses, conferences, self-education, distance learning, and e-learning [7]. Similar to other Eastern European countries, mother-and-child health was declared a governmental priority in Ukraine about two decades ago [8]. Although maternal mortality has substantially decreased in Ukraine, from 36 per 100,000 live births in 2000 to 24 in 2015 and 17 per 100,000 in 2020, it remains relatively high compared to other countries in the region [9]. Various initiatives have been implemented in Ukraine to tackle the problem. However, reducing maternal mortality, especially from preventable causes, remains on the policy agenda [6].

The aim of this article is to describe and reflect on the experience with the implementation of CME among mother-and-child healthcare providers in Ukraine. In particular, we explore achievements, challenges, and barriers in implementing modernized complex CME approaches (with international origins) in the Ukrainian healthcare context. We define CME as “the set of training activities that contribute to keeping physicians’ competencies and skills up-to-date” [10], and we refer to it as a complex approach due to the combination of formal and nonformal/informal activities, e.g., combining formal training with peer groups, conferences, etc. Thus, we analyze the case of CME among mother-and-child healthcare providers in Ukraine based on two international collaboration initiatives: the Swiss–Ukrainian program in mother-and-child health that ran from 2000 to 2015, and the more recent (still ongoing) case of the Ukrainian–Swiss project “Medical education development” implemented in 2018–2023. Taking into account the different titles of the Swiss–Ukrainian program in mother-and-child health during its various stages of implementation, in this paper we refer to it as either the ‘Swiss–Ukrainian program in mother-and-child health’ or ‘the Program’ [11]. Even though the Swiss–Ukrainian program was completed eight years ago, its unique character helps in contextualizing the complex approaches to improving the quality of maternal care in Ukraine, in particular in the area of CME. We supplement the analysis with the Ukrainian–Swiss project, allowing us to explore how challenges and barriers have changed over time. We used a case study approach and applied qualitative research methods for data collection and analysis.

The rest of the article is organized as follows. The background information and case description are presented in the following subsections, which provide details on the peculiarities of the Ukrainian healthcare system and maternal healthcare sector at the time of the Swiss–Ukrainian program and subsequent reforms. In addition, they provide information on CME in Ukraine, i.e., the system of postgraduate education that was transformed into a continuous professional development (CPD) system in 2018. The case of the two Swiss–Ukrainian collaboration initiatives is described. This is followed by the presentation of the research methods applied in the study, as well as the results of the Swiss–Ukrainian program in mother-and-child health and the subsequent Ukrainian–Swiss project “Medical education development”. Discussion and conclusions focused on complex CME approaches in Ukraine close the article.

### 1.1. Background and Case Description

#### 1.1.1. Healthcare Sector Reforms in Ukraine

Ever since 1991, when Ukraine became an independent state, socio-political and institutional changes have been taking place, with gradual and sometimes contradictory tendencies to move away from the Soviet Union structures, practices, and attitudes while trying to approach Western European standards. Many aspects of society have been impacted, including the healthcare and medical education systems. Initially, vertical institutional self-nourishing hierarchies maintained coercive and paralyzing attitudes and rules [12], which slowed down the opening of the healthcare system towards modern international approaches and technologies. The first substantial reforms in the healthcare financing system were implemented in the period from 2017–2020. The healthcare financial reform consisted of two stages. In the first stage, the focus was on primary care (2017–2019), including changes in funding mechanisms, increased autonomy for providers, and more choices for patients. In the second stage (2020), the focus was on secondary and tertiary care [6]. The reform has led to more operationalized ‘free-of-charge’ care (including universal access to obstetrical services) via the package of services funded by a single purchasing agency, the National Health Service of Ukraine (NHSU) [6,13]. This has led to improved transparency and financing of healthcare services since 2020. Although there is no empirical evidence of decreased informal patient payments and corruption due to health financing reform, anecdotal evidence suggests that providers less often request such payments due to the threat of sanction by the NHSU. Other factors include digitalization and anti-corruption measures (policies, institutions) implemented at the national level during the preceding half-decade.

Indeed, informal patient payments penetrated the healthcare system of Ukraine at most levels. On the side of the provider, informal payments led to higher income for underpaid medical staff while coercing incentives in service provision and restricting knowledge sharing within teams, which led to an outflow of patients to more competent providers (as subjectively perceived by the patients) [14]. By nature, informal payments have not been forbidden or prosecuted because the basic service package was not outlined; the state had to provide all services to everyone within the available budget [14]. From the patients’ perspective, although widespread informal payments created financial barriers to healthcare services, some patients believed that these payments were a tool that could improve the attitude and quality of individual service providers [14,15].

Apart from the health financing transformation, in 2018 the once-in-five-years postgraduate development system was changed into a more modern one linked to the CPD system of collecting points based on the training and conference activities of medical doctors [16]. Previously, the state system of obligatory postgraduate medical education received intensive criticism compared with other education programs [17]. The transformed system means that Ukrainian post-graduate medical academies or post-graduate faculties of medical universities can be providers of CPD products along with a much broader list of organizations, including those organizing international conferences outside of Ukraine.

As reported in the recent WHO country profile [6], the number of physicians in Ukraine is decreasing. Because Ukraine has only recently designed the Human Resources in Health register, the information on the number of medical doctors and nurses is not yet accurate. Nevertheless, it is already known that about a quarter of the active physicians in the country reached retirement age in 2019, and this gap has not been adequately filled by new graduates. The challenges of emigration of healthcare professionals and their seeking better-paid occupations outside the state healthcare system have contributed to this problem as well. The number of nurses and midwives has concurrently been declining in Ukraine, mainly due to “low wages, low status of the nursing profession, and limited opportunities for professional development” [6]. The healthcare labor market shortage has been especially problematic in rural areas. As a result, in addition to being underfunded and underequipped, healthcare facilities in rural areas have become understaffed.

Furthermore, human resource practices are not always transparent and provide limited educational opportunities. As noted in the literature, process quality, i.e., how care is provided, is moving ahead of structural quality [18,19]. The insufficiency of the latter, however, impedes further improvements in health outcomes. There seems to be an absence of a clear consensus about quality in healthcare, which complicates the discussion and creates obstacles to system-wide quality improvement [19].

#### 1.1.2. Challenges in the Ukrainian Maternal Healthcare Sector

After the collapse of Soviet Union, maternal healthcare services were largely underfunded (as were many other services, e.g., education and social services). Maternal care was provided at healthcare facilities (maternity hospital wards or maternity homes) that were state-owned and had line-item budgets [13,20]. After 2000, as a response to the lack of patient-centredness in maternal care, private facilities (consultation and childbirth homes) that provided expensive services became an alternative for mothers and their partners who required better services. Private maternal healthcare especially flourished after 2017. Parallel to this, perinatal centers were integrated into the public healthcare system providing services related to preterm births.

The composition of the childbirth teams has not changed over the 30 years of Ukraine’s independence: the obstetrician–gynecologist has remained the leader of the team, with a midwife as a member (who has never been an independent service provider), along with other medical specialists when relevant, such as an anesthesiologist, neonatologist, surgeon, and others [15]. Most post-graduation training (especially training provided by pharmaceutical companies) has been focused on medical doctors as a targeted audience, as medical doctors have remained the decision-makers for treatment planning and prescriptions, while midwife professional development has mainly not been tackled [15,20].

In the Ukrainian maternal healthcare sector, the delayed reforms in the healthcare system and the shortage of healthcare professionals have frequently meant inadequate care and cure, for instance due to insufficient capacity to treat severe maternal and neonatal cases. Over the years, there have been various reports on over-diagnosis and over-medicalization of pregnant women and infants with no or minor diseases, as well as iatrogenic complications through aggressive long-lasting treatments [12,21,22]. Patient-friendliness has been lacking both in theory and in practice. Individual labor rooms, partner presence during childbirth, and rooming-in were not available. It should be acknowledged, however, that recent changes such as the establishment of regional multilevel systems for perinatal care have resulted in improvements [6].

Although the health outcomes in Ukraine continue to lag far behind the European Union average, recent data on mother-and-child health reveal positive tendencies, namely, exclusive breastfeeding until six months after the puerperium has become more spread, which increased from 6% in 2005 to 20% in 2015 [13,23]. In this period, the neonatal mortality rate decreased from 9 out of 1000 to 6, respectively, reaching 5 out of 1000 in 2020 [24]. This can be attributed to the upgrading of perinatology services and innovative childbearing practices, often enabled and catalyzed by international health projects. As noticed by Lekhan and colleagues [13], “the most significant role external funding has played is in fighting infectious diseases such as tuberculosis and human immunodeficiency virus, and supporting maternal and infant health programs”. As a result, the maternal mortality indicator has improved in Ukraine for the last two decades and converged with that indicator in some other Eastern European countries, such as Hungary and Latvia, although it remains far from the level in Estonia and Poland [9].

Major national institutional achievements pairing with international initiatives have resulted, among others, in: (1) the creation of third-level perinatal centers with the aim of regionalization of perinatology services (each level takes care of sick newborns according to a well-defined potential or revealed degree of clinical severity); (2) the introduction of new registration criteria for newborns in 2007 (registration starting from birth weight of 500 g at or after 22 weeks of gestational age, as advocated by the WHO), and (3) the establishment of updated clinical protocols within national health programs and recommendations [25,26,27].

The idea of regionalizing of perinatology services was adopted from an international approach to providing perinatal services for mothers and newborns at different levels of healthcare depending on a risk assessment of the mothers’ health conditions, thereby decreasing neonatal mortality [28]. This was a way for Ukraine to achieve the Millennium Development Goals [29]. Along with updates to national legislation [27], the decision was made to set up so-called third-level perinatal centers in each region to provide highly specialized health services for mothers and newborns, with a particular focus on neonatal care for preterm deliveries. According to the structure of the healthcare facility network at that time, a third-level facility indicated highly specialized care, mainly provided in big cities.

Each of the above improvements has been assisted by international partners. A total of six international initiatives and one national project have worked for the improvement of mother-and-child health in Ukraine over the past two decades. These initiatives are presented in Appendix A.

#### 1.1.3. Continuous Medical Education in Ukraine

In Ukraine, as a former Soviet Union republic, CME has been a matter of great importance in the maternal healthcare sector. In the 1990s, many health professionals lacked information on perinatal regionalization, modern obstetrics and neonatology, and critical care approaches [30]. The concept of regionalization is used in maternal care organizations to describe the responsibilities of each level of healthcare provision, which take care of sick newborns according to a well-defined potential or revealed degree of clinical severity [31]). According to a report on governance in healthcare in Ukraine [17], CME in Ukraine was perceived by certain professionals as outdated and irrelevant. Thus, to ensure quality, efficiency, and safety for infants and parturient women, several components had to be considered, with the training of medical personnel being one of the most important. Therefore, alternative options were sought by medical doctors in Ukraine, e.g., training abroad, private training courses, or master classes in Ukraine, as well as workshops, conferences, and other events organized by third parties (pharmaceutical companies, international initiatives, etc.). However, these CPD activities were not considered formal CPD, and were not recognized by attestation committees. Modern approaches to CME superseded the previous system of post-graduate medical education.

Traditional post-graduate medical education included obligatory category improvements or recertification; every five years, medical doctors were expected to apply for a higher category delivered after points-based assessments (according to educational activities, conferences visited, etc.) and training attended, as well as exams submitted at state-recognized post-graduate institutions. According to Belli and colleagues [17], 60% of medical staff in Ukraine believed that the content of educational activities should become “more relevant to their work and fine-tuned to the needs of doctors in different specialties”. Indeed, the medical education faculties showed reluctance to shift from merely theoretical and disconnected knowledge (that frequently was not updated in keeping with recent international evidence on medical and scientific achievements) towards more modern and practical teaching processes.

Hence, until 2020 the CME and organization of healthcare service provision were characterized by top-down hierarchical decision-making and rather outdated (and most probably unsafe) medical practices. Despite the sporadic innovative approaches in healthcare services made possible by individual international collaborations, the overall environment did not facilitate local leadership for an efficient response to the multiple needs of healthcare users and medical doctors. The CPD system is now being modernized; however, because this revision is ongoing, certain aspects lack clarity, e.g., in terms the responsibility for accrediting CPD providers.

### 1.2. Two International Collaboration Initiatives

#### 1.2.1. The Swiss–Ukrainian Program in Mother-and-Child Health (2000–2015)

The Swiss–Ukrainian program in mother-and-child health was initiated in Ukraine in 1997. The goal of the initiative was to improve availability, quality, effectiveness, and access to promotional, preventive, and curative maternal and child health services in selected Ukrainian oblasts (regions), rayons (districts), and communities linked in a system of regionalization [32]. During the 18 years of Swiss international assistance, the program objectives were implemented in three principal stages. During the first stage of collaboration (1997–2000), the program focused on the provision of medical equipment to hospitals and on technological management. During the second stage (2002–2007) and third stage (2008–2015), the program extended its scope of activities and followed a complex model of CME, introduction of information and communication technologies (ICTs), and development of clinical guidelines involving multi-sectoral collaborations.

More specifically, activities on CME in obstetrics and neonatology were implemented with the collaboration and support of the Ministry of Health of Ukraine (MoH), the National Medical Academy of Post-graduate Education (NMAPE), medical universities, regional authorities, and international agencies [33]. Based on thorough preliminary and continuous assessments of clinical practices as well as on the knowledge and skills of obstetricians, neonatologists, midwives and nurses, various training activities were organized during the period 2001–2015. The training was delivered in the form of seminars, workshops, and conferences provided to professionals who were interested in the different topics. The professionals practiced different levels of perinatology. The training content was constantly adapted to specific local particularities, both clinical and structural, and particular attention was paid to the delivery of very practical and concrete sessions (clinical case discussions, skill-based training). Educational support moved from initially commonplace modalities (presentation slides, flipcharts, videos) to complex and innovative techniques (medical HiFi-simulation manikins and software).

Educational materials were developed in collaboration with Ukrainian professionals and were accredited by NMAPE, and were later introduced into teaching curricula. This aided in promoting and enforcing dissemination at a national level, eventually reaching different regions of the country that were not initially involved in the project. NMAPE supported the implementation of innovative educational approaches and institutionalized them within its curriculum of certain courses. Capacity-building regarding key mediators, such as representatives of local and regional medical and managerial authorities, was targeted through the program activities as well. Details on the Swiss–Ukrainian program in mother-and-child health are presented in Appendix B.

#### 1.2.2. The Ukrainian–Swiss Project “Medical Education Development” (2018–2023)

The Swiss Tropical and Public Health Institute was selected as the implementing agency for the “Medical education development” project. Hence, at the level of international partnership, there has been continuity in terms of lessons learned and partnership relations. Moreover, the ‘Swiss’ cross sign on medical and educational activities was already recognized in 2018 when this project started. The current project is focused on medical education and primary healthcare. The project collaborates with medical and nursing schools, building jointly with partners their capacities in eLearning, teaching excellence, curricula revision, strengthening skill labs (which are key infrastructure investments of the project), and supporting research capacity-building. On the primary healthcare level, the key areas are the advanced nurse practitioner pilot program, CPD activities for family doctors, and development of eRepository and peer groups. In addition, the project is contributing to policy-making (e.g., new CPD policies, conceptualizing human resources in the health register), experience exchange (including internationally and between educators and professionals), and communication on medical education (via podcast). Details on the MED are presented in Appendix C.

## 2. Materials and Methods

We used a case study approach as a methodology for our study. The case study approach allows focus on a particular setting or area [34]. The case study area that we explored is CME in mother-and-child care (supported by Swiss funds) in Ukraine, namely, the developments under the Swiss–Ukrainian program in mother-and-child health (2000–2015) supplemented by the Ukrainian–Swiss project “Medical education development” (2018–2023).

Different steps of data collection and data analysis were applied. First, data were collected through a review of documents within the Swiss–Ukrainian program in mother-and-child health (2000–2015). This document review employed reports of monitoring visits, observation notes, interim reports, and digests. From these documents, we extracted information relevant to the design and implementation of complex educational activities and adherence to evidence-based medicine (EBM).

Second, in-depth interviews were conducted during the closing phase of the program at the end of 2015 to explore the opinions of key stakeholders. The sample included 23 professionals, who were representatives of MoH, NMAPE, international organizations, regional decision-makers, chief medical doctors, healthcare staff, and non-governmental organizations who worked within the area of mother-and-child care. The stakeholder representatives were selected based on the method of convenient non-random sampling. The core idea of the program was presented to them by the program developers and managers through personal communication and further correspondence with the stakeholders. The participants had various job positions and levels of embeddedness in the program. We included both medical doctors who were involved in project activities and those who were not (MoH was the beneficiary of the program, NMAPE had a leading role in the program, and other international organizations and projects had more horizontal linkage with the program). Hence, triangulation of the sources of information was achieved by the representation of various stakeholders within the sample based on the notion that participants dependent on the program (e.g., heads of hospitals) may not be truly open about the program. Participants were asked to share their opinion on the program’s impact and role in mother-and-child care in Ukraine (see guide in Appendix D). The interviews were transcribed. The opinions of the stakeholders were used to illustrate the quality and impacts of program activities identified through the document review.

Third, the data extracted from program documents and the additional data collected in the in-depth interviews were analyzed by applying the method of directed qualitative content analysis (i.e., based on predefined themes). Based on Bardin’ theoretical approach [35], content analysis has three stages: pre-analysis; material exploration; and treatment, inference, and interpretation of results. The analysis focuses on discovering key elements of the study phenomenon and defining new research topics related to that phenomenon. In this study, the program-related findings were framed within the existing system of healthcare service providers on the one hand; on the other hand, they were framed within the system of postgraduate medical education in Ukraine. Both systems were closed, and jointly determined the Swiss–Ukrainian program’s activities, which were seen as responses to gaps in the systems.

Fourth, we supplemented our analysis of the CME experience within the mother-and-child program with an analysis of the experience in the more recent Ukrainian–Swiss “Medical education development” project (2018–2023). We reviewed internal project reports (e.g., annual reports, midterm reviews) and publications (e.g., conference abstracts, reports on the research conducted). This additional review allowed for updates on the perception and practice of primary healthcare providers and medical educators in terms of professional development.

It is important to note that a number of the authors of this paper are or have been managers in both the Swiss–Ukrainian program in mother-and-child health (2000–2015) and the Ukrainian–Swiss project “Medical education development” (2018–2023). This allowed for access to all relevant documents.

## 3. Results

### 3.1. Continuous Medical Education as a Core Value

Our data analysis of the Swiss–Ukrainian program in mother-and-child health showed that educational initiatives in the frame of the program aimed at setting and raising the standards in mother-and-child clinical practices both adapted to Ukrainian reality and were supported by EBM within neonatologists, obstetricians, anesthesiologist, midwives, and other professional groups less represented in the program (as shown in Table 1). The main focus was on post-graduate education and training for healthcare professionals, as these educational areas were recognized as crucial for the improvement of clinical services. Therefore, implementing CME in the frame of this program in mother-and-child health between 2000 and 2015, was materialized in a precarious educational environment trying to propose concrete answers and inputs in both clinical and in educational settings.

According to the donor policies, the program functioned in the relatively short three-year renewable phases with episodes of reconsiderations and new orientations. Additionally, adjustments to the educational models, assessments and re-assessments of pedagogical procedures, and new training tools (i.e., introduction of new teaching techniques such as medical simulations) were reasons for dynamic evolutions in educational methodologies resulting in multimodal teaching designs. However, in obstetrics and neonatology the teaching content was consistent and could be resumed for repeatedly tackling educational goals and key messages (see Table 1 for details). These objectives were based on the initial assessments of clinical knowledge and practices revealing gaps in expertise and perception of clinical techniques and attitudes.

In order to fill the gaps, the compliance and acceptance of Ukrainian professionals were absolutely indispensable in the program. Initial reluctance from program partners, such as adherence to ‘traditional’ clinical practices and unwillingness to share responsibilities and decision-making with the patients, was a fact that could easily be understood. For example, collaboration and assistance from foreign professionals might be perceived as an intrusion into local clinical life, habits, and “internal” affairs. With time and the establishment of mutual understanding and respect, confidence between partners, trainers, and trainees could be achieved. Later involvement of Ukrainian partners in training activities (training of trainers, production of teaching material and guidelines, telemedicine, medical simulation) were important steps in consolidating partnership and adherence of neonatologists and obstetricians in the program.

What has changed between the projects regarding the external stimuli for professional development? With a new MoH decree in 2018, physicians started regularly checking opportunities for CPD points collection and became more responsible for their CPD. However, during the first years of the new policies, the system has not been able to flourish due to the COVID-19 pandemic and Russian–Ukrainian war. The MoH has loosened the requirements in light of the challenging environment. Nonetheless, through joint efforts the large number of motivated medical doctors who take care for their professional development has created demand on the one hand, while on the other the small number of providers of high-quality training programs for medical doctors have received the green light for additional capacity-building with respect to their training initiatives.

Moreover, the transformation of primary healthcare through new financing principles, greater autonomy for healthcare providers, and access to public finances on the part of private organizations and individual family doctors, has stimulated the development of healthcare providers. As they have obtained more funds, they are able to pay for the services and training they need, and more motivation has been brought about by a competitive environment in which patients select their GPs on their own.

Additionally, the Ukrainian–Swiss project “Medical education development” began its educational activities on already-prepared ground, as the medical community had previously heard about the developments facilitated by the Swiss–Ukrainian program in mother-and-child health. Hence, the project had no need to explain the overall logic of the interventions despite the different target audiences (i.e., medical educators and primary healthcare providers instead of gynecologists and neonatologists).

### 3.2. Types of Educational Activities Implemented

The educational activities launched by the Swiss–Ukrainian program in mother-and-child health are presented in Table 1 in chronological order as identified in our review of program documents. Specifically, the program started with internships in Switzerland and Poland in 2001 that provided Ukrainian neonatologists with an understanding of modern evidence-based clinical practices (e.g., nursing of extremely preterm newborns). In addition, the attributes of healthcare services and atmosphere of maternity wards abroad helped to concretize the concept of patient-centered healthcare.

This educational approach impacted the increase of practical knowledge in only a limited number of professionals, and as such had a limited influence on improving the quality of work of clinical teams. Hence, in-country training for obstetricians, neonatologist, midwives, and child nurses in selected (pilot) regions was implemented during the further stages. To ensure efficiency and sustainability, teaching was later expanded to training of trainers; one team per pilot region was trained, and this team then trained other professionals. In this way, it was estimated that more than 1500 participants could be directly or indirectly trained from 2008 to 2015 in the frame of the program.

It has to be acknowledged that the development of regional trainers’ capacities was a long-lasting process that demanded regular and documented supervision. However, due to the rigid healthcare system described above, the sustainability of these teams’ work in a given environment was always at risk. For example, when national or regional power shifted after elections or for other context-specific reasons, this could affect the availability of the medical doctors involved in the teams (as loyalty to the previous authority could be seen in a negative light by the new authority).

The content of the training was developed by a team of international and Ukrainian experts, mainly obstetricians and neonatologists familiar with international medical practice and with knowledge of the English language. Professionals from medical universities, referral maternities, and regional hospitals were involved. Obstetrical modules focused on specific topics not covered by standard Ukrainian clinical protocols or manuals, i.e., multiple pregnancies or vaginal childbirth after a previous Caesarean section (C-section). Neonatology modules covered a broader spectrum of topics related to intensive care of newborns (i.e., primary resuscitation and stabilization of newborns, monitoring, ventilation support, transportation of sick newborns, nutritional issues, vertical and nosocomial infections, etc.), taking into account the major gaps in neonatal care provision described in further detail below.

Training was provided in the form of lecturing and hands-on training or articulated within the model of Ongoing Professional Practice Evaluation with concrete case descriptions, review of morbidity and mortality, discussions, and definition of lessons learned. Handouts resuming the main topics and teaching objectives were delivered. During the implementation of the training of trainers, pedagogic materials containing teaching modules, participants’ guidance, and lecturers’ manuals were developed and published (see Appendix E).

Manuals for parents and nurses were developed and disseminated among medical facilities in all regions of Ukraine. For example, pediatric nurses received handbooks containing practical issues on emergency procedures with illustrations, photos of medical equipment, advice regarding the choice and correct use of consumables, modern achievements in neonatology and intensive care, current MoH legislative documents, and principles of communication with parents of healthy and sick newborns.

*“Our training materials have several advantages over those state materials–they are evidence-based, which is new to healthcare personnel here. The training format is different from typical Ukrainian medical conferences and certificate courses because of interactive communication and the ability to actively participate in the discussion of the topics and issues. And this is very important and effective”.*

*(Chief regional neonatologist, trainer of the program)*

Particular attention was devoted to the active participation of attendants in training activities, which was regarded as crucial for the learning processes. This concept represented a rupture with previous social and educational contexts and model success situations with regard to fears of expressing own thoughts. Hence, participants were encouraged to freely express their opinions and to treat such expression respectfully.

*“The participants are often afraid to express their opinion openly, and this can be explained by the fact that they are asked their own opinion rarely, they are not sure whether their opinion is valued, they worry whether their opinion is not different from other staff or the trainer”.*

*(Regional chief obstetrician, trainer)*

In order to adapt to the working schedules of medical practitioners, short-term courses were organized lasting 1 to 2 days. Frequently, trainers visited peripheral maternity wards and assisted in clinical activities. In general, this form of training was very appreciated by the participants and perceived as concrete, useful, and effective.

*“In most areas, we employed a neonatologist and opted for short-term courses: one or two full days. Our trainers visited the area, and during the day, they worked with doctors who requested to continue this design of the training because of its perceived usefulness and effectiveness”.*

*(Regional program coordinator)*

In addition to in-class training, distance education through e-learning was introduced in order to support medical personnel at partner hospitals in their practice and to transfer basic knowledge and approaches.

*“When a person comes without adapted basic training, you have to spend a lot of time to explain the obvious principles”.*

*(Trainer)*

E-learning was recognized as a very innovative educational approach. Between 2003 and 2008, computers and internet connections were installed and program participants with computer skills had to be found and trained. While the implementation of distance learning and compliance by program partners were somewhat complex and time-consuming, they stimulated and facilitated the shift from the post-Soviet type of post-graduate medical education to a modern CME approach.

*“Now, the lexicon of the lecturers and medical doctors contains a new concept–“continuous professional development”. This is a new approach to medical education, including distance learning, telemedicine technology, new knowledge in the workplace, the emphasis on practical skills, professional competence, communication and teamwork as well as the modular approach to designing training programs and electronic textbook”.*

*(Representative of administration of post-graduate medical academy)*

CME in this program was designed for partner regions; however, educational activities covered the whole country through national conferences, guidelines and educational manuals, and courses (multiple pregnancy and vaginal childbirth after C-section, stabilization of newborn, and ventilation support of newborn) were accredited by NMAPE. Consequently, even those regions which were not directly involved in the program received program outcomes through other disseminative channels, including NMAPE.

*“First, training modules were developed by a working group of the Swiss-Ukrainian program. Obstetricians from rayon hospitals in four program partner regions received training on multiple pregnancies, and management of pregnancy, and childbirth in women after C-section. The program was consequently accredited and became the first certified postgraduate training course at the department of obstetrics at the NMAPE, which used the materials developed by Swiss-Ukrainian program in 2012. It was officially integrated in the academy’s training curricula”.*

*(Representative of post-graduate medical academy administration)*

In order to foster the introduction of developed materials into medical practice and promote administrative changes related to clinics according to priorities, regional healthcare administrators’ adherence and advocacy were stimulated by the program with the provision of parallel managerial training.

*“So as the clinical protocol does not stay in the drawers of chief doctor but worked efficiently–it is an understanding of the chief ‘what we do’, ‘why we do it’ and ‘who wants to get the result. We implement our initiatives more efficiently when “perinatal thinking” is present among heads of healthcare facilities, districts and regions”.*

*(Head of department, regional healthcare facility)*

The case of vaginal childbirth after C-section illustrates the impact of evidence-based medical practices introduced in obstetrics in 2011–2012. Courses on theory and practice implemented through the program were an eye-opener for many medical doctors, as well as for parents-to-be. This newly introduced clinical approach stipulating that natural childbirths were possible after C-section under clearly defined clinical conditions not only raised patient safety and was cost-effective, it helped to reinforce the status of mothers, as their social role might be impaired in case of non-physiological births.

*“Rural women regard a C-section as an action that indicates the inferiority of their body, as a drawback. Therefore, learning from their friends or acquaintances that they can give birth naturally after a previous C-section increases relevant requests to doctors”.*

*(Head of the maternity department)*

*“Today we are using state-of-the-art protocols for the management of pregnancy and childbirth after the previous C-section. It has been proven that assessment of risk factors, quality selection of patients and quality of follow-up, patients’ awareness and striving for success are the prerequisites of a desired outcome. And we see these outcomes ourselves. If previously we had only single cases of vaginal childbirths among women with scars on the uterus from previous C-section, then in the seven months of the current year, we have already had 30 such childbirths”.*

*(Deputy chief doctor)*

More recent experiences of the Ukrainian–Swiss project “Medical education development” demonstrate that: (a) e-learning has become widely used since 2020 due to COVID-19 related lockdowns, with some return to face-to-face offline activities after summer 2021, while the war put a pause CPD during spring 2022; since summer 2022 CPD has returned to online, though less to offline modes; (b) EBM as a concept is recognized by healthcare providers and medical educators; however, due to the lack of EBM methodology being integrated in medical school curricula and lack of English language, we observe that EBM is not yet a core principle of care provision; (c) ‘fear of expressing one’s own thoughts’ is especially noticeable among nurses during training with medical doctors, and critical comments from participants and discussions are not yet typical practice during educational activities; (d) the Swiss project’s designed and supported training and conferences are highly appreciated by participants because of the relevance of the topics, interactive approaches, practical instruments, skills which can be applied in professionally, and competence gap-driven rather than available trainer-driven approach; (e) administrative decisions on process improvement are seen as being the result of high-quality training.

### 3.3. Simulation Centers in Obstetrics and Neonatology

In the Swiss–Ukrainian program in mother-and-child health, the first training of trainers took place in 2008; since 2014, regular training and CME in the field of Obstetrics and Neonatology has been implemented for medical students and professionals. This teaching activity has enhanced interregional and international exchanges of experience through meetings, workshops, and conferences. Apart from clinical skills, the training is aimed at the development of additional professional skills such as communication and personal management.

*“Communication skills when providing care is a new and interesting topic to study and further develop health professionals of the former Soviet Union. Getting basic medical knowledge at universities and colleges, we have not received the knowledge and skills for good communication, e.g., what you say, especially if you are leading a team. The ability to hear the other person, to communicate with the patient and relatives are greatly needed”.*

*(Neonatologist)*

Establishing Simulation Centers in Ukraine went beyond the usual frames of training in mother-and-child health. This innovative concept needed special assistance in terms of practical, institutional, and economic aspects. Considerations such as infrastructure requirements, defining and procuring required equipment and technology, capacity-building, writing educational curricula, and defining its role in the Ukrainian educational context all had to be taken into account. Except in Odesa, simulation centers had not been established by state universities or hospitals because of a lack of funds. However, the manikins were not the only element of enhancing practical skills via simulation-based learning approaches. An essential role was given to higher learning and development objectives such as evidence-based methodology, scenarios adapted to the Ukrainian clinical reality, and the selection of relevant cases. Hence, after establishing four simulation centers in Vinnytsia, Volyn, Ivano-Frankivsk, and Sevastopol, particular emphasis was placed on improving capacity by employing interpersonal and group coaching as a major attribute in effective learning. A program handbook covering all these aspects was developed and made available nationwide.

Institutionalization of medical training through simulation, specifically its introduction as a mandatory step in CME and certification of medical practitioners, is ongoing. However, a number of lessons learned can be defined. For instance, the low-fidelity (simple and cheap) manikins are preferred to higher-fidelity ones. The program experience has clearly shown that the maintenance of expensive manikins places a high financial burden on the teams and their administration. In addition, institutionalization of simulation should include the development of regulatory norms that create a motivating environment for the trainers to devote their time to training courses. At the same time, clear requirements for the trainers should be agreed upon.

During the preceding decade, and with the support of the Swiss–Ukrainian program in mother-and-child health, simulation approaches have become popular in medical education. Nonetheless, those who launch simulation centers and skill labs are more focused on infrastructure and manikins than on trainers’ competencies (including an understanding of debriefing), revision of medical students’ competencies, the skills to be a part of skill labs training, and their integration into the curriculum. Hence, the Ukrainian–Swiss project “Medical education development” is not only supporting the development of existing skill labs or new ones, it is concentrating on the professional growth of skill lab trainers and their skills in scenario development and assessment. Presently, policymakers are discussing certification of skill labs and simulation centers to ensure their adequate functioning and the maintenance of national standards.

### 3.4. Monitoring and Evaluation Visits

Our review of documents from the Swiss–Ukrainian program in mother-and-child health showed that monitoring and evaluation were important supplementary activities to the training delivered. Follow-up visits were conducted in order to consolidate knowledge and skills gained during theoretical and practical training and to facilitate their implementation. The monitoring scheme included several follow-up visits that were initiated about ten weeks after the training. The follow-up visits usually happened at 6-month intervals. A focus was placed on the evaluation of general factors affecting hospital performances and bringing to light the problems faced by health professionals in the provision of perinatal care, as well as identifying solutions with clear guidelines for action.

*“Some doctors are already responding constructively. They say that after monitoring visits, they want to change something–do differently and better. I would not say that the attitude towards monitoring visits is 100% positive in hospitals, and probably absolutely positive perception will never be reached. But the perception that “the chief will come and punish” is not already there. In contrast, most teams say that these visits are an opportunity to have “a fresh view” in terms of where and how you work–as it is difficult to objectively assess the reality when you do the same from the morning till the night”.*

*(Regional chief pediatrician)*

The program monitoring visits used to be agreed upon two weeks in advance with the administration of the healthcare facilities. Monitoring teams consisted of neonatologists, obstetricians, and/or pediatricians, usually leading regional professionals. They assessed the situation through observation of work and interviews with health professionals and child-bearing women. The visits corresponded to a new horizontal reciprocal exchange of information and expertise, increasing the chances for further dissemination and implementation of the issues addressed during training seminars. Moreover, the program introduced the practice of inter-regional visits, which was accepted positively and implemented by the program teams.

*“Monitoring visits–when the teams from other regions came to our region, and vice versa–were a unique opportunity to see how other doctors work and what methods and achievements they have. You notice many positive aspects and then you bring these positive insights into your medical institution. Also, after the seminar on the principles of the monitoring visit, I observe that the attitude of medical doctors towards monitoring has changed. Each next visit is conducted much easier”.*

*(Regional chief obstetrician-gynecologist)*

Monitoring visits on the part of training providers remain a rare practice. From the perspective of the Ukrainian–Swiss project “Medical education development”, such visits seem to be more relevant in the case of practical skill development of healthcare service providers and less feasible for medical educators (as after the syllabus, i.e., the topics of classes and assessment plans, is developed, education has fewer practicalities and changes require more time), in addition to being less functional in revealing changes in primary healthcare facilities due to the distracting matters of uncertain environment and lack of physical safety. Priorities in service provision seem to be more focused on staff retention and mental health support rather than new developmental challenges. Nonetheless, in this difficult context, reflection on the practices and changes is helpful. Student or patient feedback is helpful to review what has changed after the training, as is feedback from providers and trainees (educators or physicians/nurses). For example, in the Ukrainian–Swiss project “Medical education development”, nurses who extended their roles and competencies in service provision could recognize the pre-heart failure condition after recent ECG training, and shared this achievement with the training organizers.

### 3.5. Changes in Infrastructure and Upgrading of Medical Equipment

The Swiss–Ukrainian program in mother-and-child health placed special attention on integrating training activities in the context of poor healthcare infrastructure, and as such it concentrated on upgrading facilities and procuring essential equipment. Maternity and neonatology wards and neonatal intensive care units (NICUs) of third-level referral centers and second-level regional health structures were involved in facilitating the creation of a functional network with basic minimal infrastructure for perinatal regionalization. Medical communities and local authorities were involved in defining needs, participating financially, and organizing the reconditioning of the premises. Special training on fundraising strengthened the capacities of head doctors and administrators in negotiating state and private funding. Partnerships between government bodies, non-governmental agencies, and private organizations in financing the development of health services were created.

*“The recommendations of the program experts who came to our institution helped very much for proper zoning premises, so, we did everything correctly from the first attempt. The local budget funds were allocated to buy a part of the necessary medical equipment, and then our institution was officially included in the program, and the program provided the rest of the equipment. Nowadays, our maternity ward and intensive care unit are well-equipped with everything we need”.*

*(Chief doctor)*

Renovations of buildings and facilities aimed to create patient-friendly premises. New spacious, comfortable, and bright obstetric wards and patient individual or two-bed rooms were set up with the aim of closeness to medical facilities such as operating theatres and NICUs. These fitting-outs created space for a partner to be present during childbirth, rooming-in, skin-to-skin policies, and the introduction of “responsible parenting schools”, as well as for “open days” at the hospital.

*“The real innovation is the Open Day in the maternity hospital–each Saturday, from 10.00 to 12.00 the couple can visit the maternity ward, its atmosphere and conditions where they will give birth to their baby. Doctors believe that this promotes better psychological preparation for childbirth”.*

*(Obstetrician)*

*“When school started, we did not expect high attendance, but now we have to work in several shifts because the demand is very high. Most pregnant women come with partners and we are preparing them for the process of birth. You will not believe it, but in 2007 we had no partner births in our hospital, and already in 2013, the rate increased to 82%”.*

*(Head of department at healthcare facility)*

Value-focused locations have been a focus of the Ukrainian–Swiss project “Medical education development” from 2020–2021, e.g., student-centeredness in medical education should be supported by student spaces (hubs) in which students have the opportunity to discuss their ideas, develop projects, and organize informal or additional non-formal educational activities. Such infrastructure developments support the overall strategic values of being client-oriented, and are one of the distinctive features setting the present approach apart from the Soviet approach to education (teacher-focused) and healthcare (doctor-focused). Unfortunately, the reduction in the financial capacity of partner and the damage to infrastructure due to the war have negatively impacted such infrastructure projects designed earlier; however, new construction and reconstruction of hospitals and schools are taking place even during the war.

### 3.6. Changes in Clinical Practice of Mother-and-Child Care

According to the documents of the Swiss–Ukrainian program in mother-and-child health, the progressive advance of practitioners in terms of knowledge and awareness toward international standards could be clearly observed. Among other examples, we note routine continuous monitoring policies in NICUs (such as determination of oxygen content in blood with pulseoxymeters, cardiac monitoring with electrocardiogram, and registration of respiratory rate connected to adequate alarm systems for early detection of anomalies). Another example is the improved capacity provided for ventilatory assistance of sick newborns, whether invasive or non-invasive (i.e., intermittent mechanical ventilation, nasal continuous positive airway pressure or bilevel positive airway pressure). Protocols for the management of multiple pregnancies, primary resuscitation of newborns, prevention and cure of Hyaline Membrane Disease, in-utero transfer, etc., have begun to be routinely implemented. Out of all educational and connected initiatives that contributed to the changes in clinical practices, the main reason for these improvements can be attributed to CME. It is certain that this educational activity has met the needs of clinicians and has helped substantially in improving practical skills as described. Practitioners were unanimous in acknowledging that the new training experiences were helpful in their practical work and that they would certainly recommend the training course to their colleagues. Mothers saw positive changes in the attitude and in the clinical recommendations they received in peripartum.

*“There are the same people working here, but their attitude towards the patients, as well as their practices, has changed a lot”.*

*(Mother)*

*“Thanks to our joint efforts in collaboration, we achieved a lot, for example, the loss of children up to 1 year of life (particularly early neonatal age) has significantly decreased. And I can say that this figure is 90% possible because of the training and improved knowledge and skills of medical staff”.*

*(Regional chief neonatologist)*

### 3.7. Obstacles Yet to Be Considered

We found during the document review that the obsolete system of healthcare service provision in Ukraine represented an important reason for the slow pace of healthcare improvements. Obstacles included poor financing that led to the lack of medicines, equipment, and consumables; inadequate human resource development and the outdated official system of CME; and rigid management practices, including in the area of quality improvement. Other barriers that the program had to take into account during training and coaching activities were poor knowledge of the English language among the medical and paramedical professionals, which sometimes, especially initially, led to a reluctance to open up to new advice and international standards.

A general atmosphere of fear generated by a vertical long-distance hierarchy (chief’s or professor’s opinion, “clinical expert commissions”, and MoH audits) contributed to these attitudes. Superiors in Ukraine were often disconnected from clinical reality, and this might have increased over-diagnosis and over-prescription as a mechanism of self-protection on the part of practitioners.

*“The approaches offered by the program have not resonated immediately with our doctors because all medical personnel used old approaches, and it was important first to prepare the personnel psychologically for the introduction of new perinatal technologies–to change the usual vision and to learn a lot. Later, during the training and visiting activities, doctors realized that these methodologies and practical skills were really effective, which in turn gave them more motivation to learn and to change”.*

*(Chief doctor)*

*“Not all medical professionals want to change their old stereotypes of pregnancy and childbirth”.*

*(Medical doctor)*

It was recognized that the implementation of perinatal regionalization and its network needed further improvement. Scarce institutional commitment and the problematic financial situation hindered the adequate allocation of means for this purpose (transportation in equipped ambulances, state of the roads, helicopter transfer, etc.). More quotations that illustrate these statements are presented in Appendix F.

On the clinical level, less encouraging results compared to the achievements already described concerned the understanding of vertical infectious diseases (transmission from mother to baby, mainly in peripartum) and especially the misuse of antibiotics, which are prescribed excessively and not in line with international recommendations. This can be imputed to the insufficient capacities of microbiological laboratories to identify pathogens (i.e., frequent positive Coagulase-negative Staphylococci of uncertain interpretation, and rare positive findings of Group B Streptococcus) and the myth of antibiotics being a prophylactic guarantee against (nosocomial) infections. Further training efforts are advisable. In addition, nutritional problems continued to subsist due to little availability of adapted consumables (central venous catheters for adequate parenteral feeding) and enteral feeding formulas.

With the recent launch of the NHSU and new healthcare financing principles, healthcare facilities have more available funds and materials. Chronic underfunding has been responded to by these reforms; however, the war has led to lower fiscal capacity on the part of the state. What remains among the achievements are new and more efficient mechanisms of state funding distribution and its relevance to service provision as opposed to simply maintaining beds. New service provision policies require new and more extended competencies on the part of healthcare managers of autonomous facilities. A lack of studies limits the understanding of both areas. Because directors of healthcare facilities must deal with NHSU service packages, funding distribution, and related policies, this may be a sign indicating that managerial knowledge and skills are developing as well.

## 4. Discussion

The results presented above show how complex CME approaches (with international origin), specifically those of the Swiss–Ukrainian program in mother-and-child health, have been applied in the Ukrainian context. The fresher lens of the Ukrainian–Swiss project “Medical education development” can supplement and update the findings of this program. While the implementation of these two international collaboration initiatives has had multiple benefits for the Ukrainian healthcare sector, it has faced various challenges and barriers as well. Below, we discuss these findings in light of the specific healthcare and education systems in Ukraine and the healthcare labor market shortages outlined at the outset of the article.

### 4.1. Innovations in CME and Clinical Practice

Innovative education programs are required to ensure the necessary competencies of the healthcare workforce for tackling the current health risks [1]. In particular, in maternal healthcare, CME could help to enhance the skills of healthcare professionals, improve medical quality, and increase women’s satisfaction with service use [3]. It is especially crucial to link CME, as well as undergraduate training, with practice in order to define problems and gaps in practice that can be addressed through educating staff [36,37]. This has been a guiding approach for designing the activities of the Swiss–Ukrainian collaborations analyzed in this article.

As shown by our results, during the 15-year Swiss–Ukrainian program in mother-and-child health, clinical changes could be witnessed at many levels. Prior to program implementation, assessments clearly found a lack of awareness and expertise on the part of clinicians, as well as a lack of modern equipment [38,39]. Importantly, the concept of clinical monitoring [40] was not perceived as crucial, frequently leading to instantaneous instead of contextual diagnosis while missing out on many situations in which emergency handling could be life-saving (as demonstrated by our results). Gestational age (pregnancy term) was often defined inaccurately. Adequate monitoring of the fetus’ well-being during pregnancy and birth was lacking.

As described in the results section, the Swiss–Ukrainian program in mother-and-child health facilitated the rapid implementation of highly innovative simulation medical education that allowed Ukrainian medical doctors to avoid the thorny approach of trial and error, and provided an impetus to the further development of education scenario development. In addition, the program positively impacted the clinical safety of childbirth, and specifically the core concept of service quality. Simulation in medical education, which is a gold standard in many modern teaching institutions worldwide [41,42,43], became possible in Ukraine due to this program. It helped in strengthening practical clinical skills, promoting teamwork in multidisciplinary teams, and improving communication prowess. As a result of the availability of simulation centers developed within the program’s sustainability strategy, in the year after the program ended the trainers continued to share knowledge with new groups of practitioners, developing new scenarios [44] and acknowledging their achievements by the international scientific community. Moreover, the national collaborators of the mother-and-child health program have continued their role in the Ukrainian–Swiss project “Medical education development”. It is indeed very helpful to have a ‘simulation school’ available for deployment of new initiatives.

However, chronic shortages in funding and a lack of experienced EBM staff make it difficult to install new simulation centers in Ukrainian educational and medical institutions. As mentioned earlier, the lack of necessary investment undermines the quality of healthcare provision and increases the healthcare labor gap in Ukraine as well as in the Eastern European region more generally [2,6]. This case study shows that international projects can play an important role in securing such investment and improving quality by bridging clinical practice and education, including the development and implementation of clinical guidelines.

### 4.2. Motivation of Healthcare Providers

Healthcare professionals in the Eastern European region often lack motivation due to unsatisfactory working conditions and low reimbursement rates [2]. Underfunded and underequipped facilities coupled with overburdened and underpaid staff leave no incentives for quality improvement [3]. Ensuring better conditions at healthcare facilities and increasing patient comfort brings about better work satisfaction for medical staff, which is a key motivational job-related factor [45], as reported in the present article.

Our results describe numerous interventions in healthcare provision, including reorganization of the maternal department space, monitoring visits, and more patient-oriented approaches. Indeed, the attention paid to professionals (via international expertise), considering and responding to their professional needs, and development of interpersonal skills (e.g., communication) and networking have become crucial in establishing a trustful and safe environment where changes in clinical practice have been possible [46].

Before the Swiss–Ukrainian program in mother-and-child health, over-medicalization within care for women and newborns was widely spread. No individual birthing rooms were available; several women were sometimes giving birth in one room at the same time. Partner presence during childbirth and rooming-in was not allowed; newborns were kept all together in separate rooms. Frequently, nutritional needs essential for the healing of sick newborns were not met. Overall, the care provided to sick neonates was unsatisfactory [12,38].

Most of these practices have become remnants nowadays due to this program, which has improved mothers’ childbearing experience, as illustrated by our case study. More trustful relations between healthcare providers and families can have a large contribution to service providers’ job motivation as well. Moreover, the program has helped to develop communication skills, which contribute to establishing a trustful and safe clinical environment [46]. Thus, this case study demonstrates the importance of the development of interpersonal skills in enhancing the experience of both patients and service providers [4]. Improving the job satisfaction of healthcare professionals in Ukraine and in the Eastern European region more generally could be an important factor in motivating health service professionals to remain in practice and discouraging labor migration.

### 4.3. Promoting Openness, Transparency, and Integrity

One of the major obstacles to service improvement in Ukraine, as in other Eastern European countries, is the lack of financial incentives for individual healthcare providers [13]. Recent reforms in Ukraine have resulted in increased salaries of healthcare professionals and better incentives for providing services at the primary level [6]. Nevertheless, chronic shortages and lack of leadership and transparency constitute a situation where healthcare service providers are underpaid and a major part of income (especially for surgeons and gynecologists) is earned informally. In particular, for decades the salary of medical doctors has been lower than the industrialized world average. Therefore, under-the-counter payments paid by healthcare users are appreciated [6,15] and have become part and a parcel of the system of healthcare financing in Ukraine and elsewhere in Eastern Europe. These informal honorariums do not simply maintain the living standards of the healthcare providers; they are invested by physicians in their education (especially in the development of practical skills) and in the healthcare facilities where they work [17]. Indeed, Ukrainian hospitals rely on both informal and quasi-formal payments, as they supplement poor public funding and are typically not audited [17].

The Swiss–Ukrainian collaborations, while bringing forward innovative CME and EBM practices, also promoted the values of openness, transparency, and integrity. This is illustrated by the findings of this case study. In the Ukrainian healthcare sector, strong formal and informal parallel structures exist in virtually all areas, including education [47]. This setup penetrates Ukrainian heath care because informal patient payments are almost the sole motivation for healthcare providers [48], and as a result clinical and organizational practices cannot be changed solely by the introduction of new rules and policies. New ways of doing things in healthcare might be possible if healthcare providers find the practice useful for retrieving additional formal income. Because the Swiss–Ukrainian program had clear policies of transparency and accountability, which conflicted with the Ukrainian healthcare sector practice, it was necessary to find a compromise. Modern equipment is one way to address both formal and informal objectives: typically, healthcare providers cannot afford to procure expensive equipment, which is linked to upgrading the quality of services and safety of care; thus, the Swiss–Ukrainian program in mother-and-child health entered the facility through the ‘equipment door’ (which is seen as a good base for generating more payments), subsequently entailing the interest of health service providers to many other service and clinical practice improvements.

The Swiss–Ukrainian collaborations presented in this article are examples of how international projects can contribute to improving the managerial capacities of facility leaders through training and plans for facility development. In fact, chief medical doctors are advised to supplement such collaborative input with funds raised from other sources, e.g., public–private partnerships. Thus, the Swiss–Ukrainian collaborations have indirectly contributed to awareness of the importance of transparent policies in service provision on the part of healthcare staff, as well as the development of fundraising skills and project-based management of healthcare facilities. This example could be helpful for other Eastern European countries where openness, transparency, and integrity need to be promoted among the healthcare workforce.

### 4.4. The Sustainability and Impact of International Assistance and CME

Ukrainian mother-and-child services previously had no tradition of CME. The CME philosophy was hardly even accepted at the beginning of the Swiss–Ukrainian program; however, in spite of the need for complex and integrated activities, this has become possible, as suggested by our findings. A complex approach in CME as a vital step towards better quality and access shows its sustainable nature [49]. First, the custom of professionals to update their skills and knowledge has been cherished during the Swiss–Ukrainian program, and the need for professional improvement has been actualized. Second, trainers and trainees from both clinical and university settings participated in program activities, which ensured the wider dissemination of knowledge and practices (vertically, i.e., in the curricula of post- and undergraduate education and horizontally, i.e., professionals from other clinical areas). Third, improved communication skills have become a ground for a more intensive collaboration with colleagues and the sector in general (sharing the experience and practices, initiating changes) as well as with the public and patients. However, after the end of the Swiss–Ukrainian program in mother-and-child health the scope of activities became narrower because of the unavailability of donor input in the further development of obstetrics and gynecology, e.g., fewer conferences, workshops, and direct assistance from international experts. Nonetheless, this program model represents an excellent opportunity for local leaders to take responsibility for maternal care by following the successful pattern of collaboration in responding to newly emerging needs.

Importantly for Ukraine and other Eastern European countries as well, clinical and educational solutions cannot be directly transferred from one international project in one country to another. The priorities have to be (and were) defined according to the population’s health needs, feasibility, and local capacities [50]. To provide an extreme example, it would have been inappropriate to train a large number of Ukrainian practitioners on coelioscopic procedures for newborns. On the contrary, training on promoting breastfeeding alone would have been too simplistic. Thus, capacity building on handling sick newborns and preterm babies based on adapted intensive care methodologies in the Ukrainian context was realistic, feasible, and useful in terms of perinatal morbidity and mortality, even though the approaches were complex and multimodal. The latter approaches of necessity spread to other issues, whether “simplistic” ones (breastfeeding, skin-to-skin) or more complicated ones (surgical procedures).

As mentioned in the case description and explained in the results, the length of the program was almost two decades, which played a key role in achieving a real shift in the clinical practices and engagement levels of all stakeholders. One of the main lessons learned was the importance of the time factor; in order to allow efficient clinical implementation of complex CME in post-Soviet countries, a minimum period of 10 to 15 years has to be allocated [51]. In this case, awareness and adherence were stimulated within this time frame, passive and active learning processes were integrated, and new concepts became real and concrete and could be tested in clinics. In addition, the passage of time helped new generations of clinicians to appear and new attitudes around teamwork to be created.

### 4.5. Study Strengths and Limitations

This study has disseminated the experience with CME in Ukraine, and specifically highlighted the benefits and challenges of the Swiss–Ukrainian collaboration. The main strength of this study lies in its analysis of long-term collaboration focusing on the healthcare environment and the complexity of the activities that bring about change in both the de jure and de facto dimensions. However, this study is not without limitations. As mentioned in the methods section, some of the authors are or have been managers in the two initiatives studied. While this ensured access to all relevant documents, it could have caused researcher bias. However, not all authors were involved in the programs, which means that the possibility of such bias has been reduced.

Notably, the lack of a proper nationwide monitoring and evaluation system makes precise quantitative estimations involving the Swiss–Ukrainian collaboration impossible. Nevertheless, the study has generated evidence on the experience of this collaboration and the perceptions of stakeholders. In addition, the experience analyzed in this case study tackles several regions which were the focus of the program. Although the Ukrainian healthcare system is essentially centralized de jure, de facto there are substantial differences across the regions because of different historical, economic, and socio-cultural aspects. Prior to the healthcare reforms, obstetrics and gynecology were very specific areas of healthcare service provision having, e.g., higher status of healthcare service providers and more resources compared with primary healthcare [52]; therefore, the motivation and context of other service providers could be slightly different. Another issue is that the number of participants and professional groups involved in the study could have been higher, as the Ukrainian context is not homogeneous in terms of maternal healthcare practices. Although there is a high degree of uniformity in terms of perceptions, we might have missed important ‘islands’ in this case study. Finally, because we were not sure about the degree of openness of the participants, we involved various groups, including those less dependent on program activities (e.g., international organizations) as well as other modes of data collection (e.g., observation).

Thus, the results must be transferred to other sectors and locales with caution and while ensuring careful understanding of differences in the sector features. For example, due to the latest health financing reforms the status of primary healthcare physicians has been substantially increased by financing, communication campaigns, and the organizational performance of primary healthcare centers.

## 5. Conclusions

Based on the results presented in this article and their discussion, and considering our study aim, we are able to formulate several key conclusions regarding the experience with the implementation of complex CME approaches in Ukraine. We conclude that complex approaches to implementing CME, namely those in the Swiss–Ukrainian mother-and-child health program, have had positive effects according to the stakeholder representatives involved. Therefore, the experience of this program should be considered in further initiatives in the area of mother-and-child health. An example of such initiatives relates to the implementation of similar activities through the new Ukrainian–Swiss medical education development project.

The effect of the numerous multi-level educational activities designed to change medical practice and patients’ health outcomes is especially visible in a long-term perspective [53]. Times when one or two post-graduate courses were enough are already in the past. A personal life-long educational trajectory with formal and nonformal/informal education is becoming a reality for healthcare professionals [54,55]. However, this creates challenges around assessing the impact of education on professional practice and growth, especially if it is conducted immediately after training, conferences, and meetings with peers. Longitudinal data could be supportive of understanding the impact of educational activities. Nonetheless, in resource-poor settings and those with war, it requires extraordinary effort to invest in this kind of evidence collection.

As shown in this study, the program activities in the Swiss–Ukrainian program in mother-and-child health were designed in view of the relatively weak institutional and technical capacities of the county, i.e., post-Soviet mentalities and an unstable political climate in a societal transition period. Despite the number of problematic areas in the Ukrainian healthcare sector, including the lack of funds, their inefficient allocation, constant changes of authority, and an unstable political situation, the program led to the introduction and adaptation of international practices, assuring its sustainable presence in the service provision.

The experience of implementing this program clearly shows that the contextualization of educational activities has to be taken into account. The recent experience of the Ukrainian–Swiss project “Medical education development” implemented under the COVID-19 pandemic and war–related restrictions demonstrates that difficult contexts may bring about new insights and foster new practices (e.g., e-learning during the COVID-19 pandemic and Eurointegration in Ukraine during the war).

This study concludes that a shift from the old and often unsafe clinical practices to evidence-based and efficient mother-and-child care can be realized in the post-Soviet settings notwithstanding poor governance. A complex CME approach based on the values of openness, respect, dialogue, and professionalism can be successfully implemented by an international assistance program despite the resistance of the system towards change.

In Ukraine and other Eastern European countries, such initiatives are of crucial importance for ensuring necessary competencies on the part of healthcare professionals, enhancing their job motivation, and improving the quality of healthcare provision. Investments in infrastructure and life-long learning programs can help to train and retain healthcare professionals in national healthcare systems by providing better work conditions and opportunities for professional development.

## Figures and Tables

**Table 1:** Content and scope of the Swiss–Ukrainian collaboration in mother-and-child health.

Type of Activity	Target Audience	Topics	Time Frame
Educational activities for medical personnel			
Internship abroad (in Switzerland and Poland)	Neonatologists of regional hospitals from pilot regions	Neonatal intensive care	2001–2004
Direct training by foreign experts	Obstetricians, neonatologists, anesthesiologists, midwives, child nurses from regional and district hospitals in selected regions	Management of pregnancy, incl. 1st trimester ultrasound, gestational diabetes, pre-eclampsia, dangerous signs, etc. Complicated childbirths: breech position, induction, Cesarean section, etc. Basics of neonatology, incl. newborn physiology, pathophysiology, modern therapeutic approaches on primary resuscitation, ventilator support, the preterm, monitoring, nutrition, nursing, iatrogenic complications, vertical infections, clinical case descriptions and discussions etc.	2002–2007
Training on medical equipment	Medical personnel of partner hospitals that received the equipment from the project. Equipment for antenatal care and labor monitoring, primary resuscitation and intensive care.	Use and maintenance of equipment; mostly held together with a representative of the supplier	2003–2013
Training for trainers and cascade local training	Local training teams; obstetricians, neonatologists, Anesthesiologists, midwives, child nurses from regional and district hospitals in selected regions	Antenatal care; Multiple pregnancy and childbirth; Vaginal childbirth after previous Cesarean section. Stabilization and transportation of newborn; Breathing support to newborns.	2008–2015
Distant education			
Distance lecturing via IT solutions	Medical personnel of partner hospitals from project regions	The same topics as for local training; also, topics defined additionally by local teams	2003–2015
Electronic manuals for combined courses and self-learning	Obstetricians, midwives, neonatologists, child nurses.	Management of normal pregnancy; multiple pregnancy; shoulder dystocia; primary resuscitation; continuous positive airway pressure	2007–2015
Conferences			
National level	Obstetricians and neonatologists from all over Ukraine	Multiple pregnancy; preterm newborns; annual conferences of Ukrainian professional associations	2007–2015
Regional level	Medical personnel of district and regional hospitals from project regions	Antenatal care; transportation of pregnant woman; primary resuscitation; transportation of newborn	2012–2015
Simulation education			
Establishment of regional Simulation centers	Four training teams, consisting of obstetricians, neonatologists, anesthesiologists, midwives, nurses, IT specialists. The teams provide training for medical personnel of project and neighboring regions.	Emergencies in obstetrics and neonatology. Combination of practical skills and teamwork, communication, and debriefing.	2013–2015
Various activities linked to project educational component			
Procurement of essential medical equipment	Medical personnel or obstetrical and neonatal units	USD, CTG, continuous positive airway pressure, pulse oximeter, phototherapy lamp, etc.	1998–2015
Formative supervision	Medical personnel of partner hospitals and its’ management.	Monitoring and support of the implementation of new practices after training.	2008–2015
Development and introduction of clinical guidelines	Obstetricians, neonatologists, anesthesiologists, midwives, nurses	Transportation of pregnant woman; multiple pregnancy and childbirth; primary resuscitation; transportation of newborn.	2011–2015

**Figure A1:**
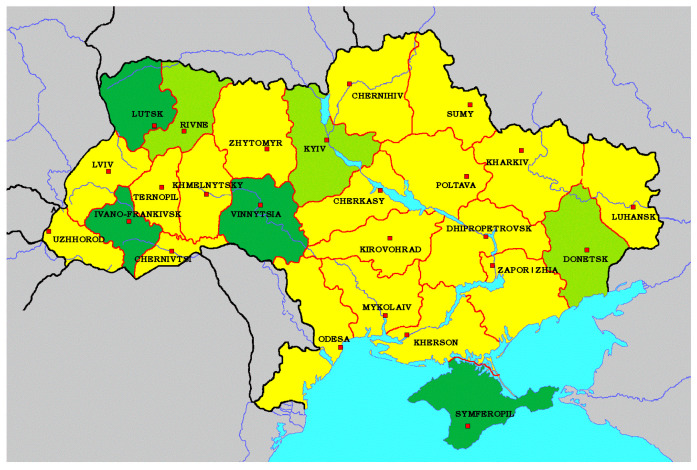
Geographical coverage of the Swiss–Ukrainian collaboration.

## Data Availability

The data are not publicly available due to confidentiality issues.

## Abbreviations

CME	continuous medical education
CPD	continuous professional development
C-section	Caesarean section
EBM	evidence-based medicine
ICT	information and communication technologies/information technologies
MoH	Ministry of Health of Ukraine
NHSU	National Health Service of Ukraine
NICUs	neonatal intensive care units
NMAPE	National Medical Academy of Post-graduate Education, Ukraine
SDC	Swiss Development Cooperation
WHO	World Health Organization

## Appendix A. Seven International and National Initiatives Which Have Worked for the Improvement of Mother-and-Child Health in Ukraine (Alphabetical Order)

Canadian educational initiatives (funded by the Canadian Government, managed by the University of Alberta; 1993–1999), organizing assignments of Canadian pediatricians in Ukrainian hospitals and providing internships for Ukrainian medical personnel in Canadian hospitals with focus on pediatrics and intensive care.

German–Ukrainian project in obstetrics, perinatology, and neonatal medicine (funded and implemented by the German Federal MoH and Embassy of German Federal Republic in Ukraine, 2012–2015), providing series of training for medical personnel of newly established perinatal centers as well as internships in German hospitals for several Ukrainian healthcare professionals.

Mother and Infant Health Project (USAID and private funding; 2002–2010; 20 maternity hospitals in 12 regions of Ukraine), focusing on the implementation of partner presence during childbirth, avoidance of unnecessary C-sections, amniotomies and episiotomies, free positions during birth, immediate skin-to-skin contact, early breastfeeding, rooming-in of mothers and newborns, and training of medical practitioners in project pilot settings. A package of essential perinatal practices, including evidence-based practices throughout Ukraine, was implemented. In addition, basic equipment was provided to the project facilities through private funds.

The New Life national project was launched in 2012 on the President’s initiative. It aimed at reducing maternal and perinatal mortality via regionalization of perinatal healthcare. The main project task was to establish modern perinatal centers at the level of main regional cities to provide healthcare for pathological pregnancies and deliveries and for preterm and sick newborns.

Swiss–Ukrainian collaboration in mother-and-child health: upgrading perinatology health services (Swiss funding and regional state and private financing; 1997–2015), implemented in about 90 maternal departments/hospitals in five regions (oblasts) of Ukraine. The specific characteristic of these activities was their complex, multimodal, and integrated approach.

UN Agencies (UNICEF, UNFPA, WHO): support for policies of dialog in mother-and-child health in Ukraine by promoting certain practices (breastfeeding, vaccination, etc.) and the Baby-Friendly Hospitals initiative, introducing modern auditing approaches (“Beyond the Numbers”), and organizing respective training for medical professionals. Information on the features of the Baby-Friendly Hospitals initiative is available at http://www.unicef.org.uk/BabyFriendly/News-and-Research/Research/Baby-Friendly-Initiative/, accessed on 25 June 2023.

## Appendix B. Swiss–Ukrainian Program in Mother-and-Child Health

The Swiss–Ukrainian collaboration in mother-and-child health was established in Ukraine in 1997. Several phases of this collaboration took place.

Stage 1. Neonatal project (August 1997–March 2000): upgrading birth clinics (mainly equipment) and intensive care departments. Donetsk, Volyn (Lutsk), Ivano-Frankivsk, and Rivne regions (141 hospitals). Swiss Economic and Cooperation Development (SECO) funding.

Stage 2. Improving perinatal health services project (phase I: January 2002–December 2004; phase II: January 2005–December 2007): clinical guidelines development, health promotion, capacity building, introducing modern public health concepts and health management tools. Donetsk, Volyn, Ivano-Frankivsk, and selected institutions in Kyiv. Swiss Development and Cooperation (SDC) funding.

Stage 3. Swiss–Ukrainian Mother-and-Child Health Program (Phase l: January 2008–April 2011; Phase II: May 2011–April 2015; SDC): improved availability, quality, effectiveness, and access to promotional, preventive, and curative maternal–child health services in selected Ukrainian oblasts, rayons, and communities. Volyn, Ivano-Frankivsk, and Vinnitsia regions and AR Crimea (until April 2014); in total, 82 rayon-level and 8 oblast-level institutions.

The related projects and activities are presented in the table below.

**Table A1:** Projects and activities related to the Swiss–Ukrainian collaboration in mother-and-child health.

	Project Name	Period	Intervention Areas	Components
1	Neonatology Project	8/1997–3/2000	five oblasts (Rivne, Volyn, Ivano Frankyvsk, Donetsk, Kyiv); Equipment to 141 facilities	Training courses to accompany the introduction of new equipment
2	Perinatal Health Programme	10/2001–9/2004	five oblasts (Rivne, Volyn, Ivano F, Donetsk, Kyiv); mainly oblast-level interventions	Perinatal service strengthening in five oblasts; capacity building for one perinatal centre (Volyn)
3	Mother and Child Health Programme, Phase 1	1/2005–12/2007	Health Promotion (Donetsk) Perinatal Services (two pilot rayons in Volyn; two pilot rayons in Ivano F.)	Integrated Perinatal Services Intervention guidelines and training, incl. health promotion
4	Mother and Child Health Programme, Phase 2	1/2008–04/2011	Oblasts: IF, Volyn; Crimea; Vinitsa (approx. four rayons per Oblast).	Upscaling of four models developed in previous project
5	Mother and Child Health Programme, Phase 3	05/2012–04/2015	Oblasts: IF, Volyn; Crimea; Vinitsa (all 71 rayons included).	Infrastructure upgrading, continuous education courses, health informatics, service provision re-configuration; Medical Simulation Centres as innovation.

Program components: maternal-child health promotion; integrated perinatal care model; information and communication technologies; management capacity.

Key implementation partners: Ministry of Health of Ukraine (MoH); National Medical Academy of Post-graduate Education (NMAPE); Regional and local health administrations and healthcare institutions; medical universities; WHO, UNICEF, UNFPA; National Project Nove Zhyttya (New Life Project, NLP); projects funded by other donors (JSI/USAID).

The primary beneficiaries are indirectly sick newborns and mothers in the target regions/districts and secondarily those who are in need of improved mother-and-child health services: (a) men and women in the reproductive age group; (b) couples or single women expecting children; and (c) newborns and infants. Project activities target capacity building of key mediators: (a) representatives of local and regional authorities and (b) medical and managerial staff of healthcare institutions (referral oblast maternities and pediatric hospitals, central rayon maternities, women outpatient clinics, rural ambulatories, family planning centers, others).

Implementation strategies of Stage 3 (2008–2011, 2011–2015):

Teaching, training and continuous medical education: training of medical staff, establishing or strengthening of regional training teams; training for healthcare managers; development of new teaching materials and anchoring previously and newly developed teaching materials; training of trainers; improving continuous education by introducing new concepts, i.e., internships, simulation education, eLearning, telemedicine, etc.

Capacity Building on Concepts and Management Tools: formative supervision (monitoring experts); Perinatal Service Package (approved by the Ministry of Health (MoH for nationwide dissemination); ICT based products (telemedicine platform; Perinatal Registry; Medical Equipment Information System; eLearning modules), regional e-Health strategy development.

Capital Investments: physical rehabilitation of facilities; essential equipment purchase; IT infrastructure.

Content Development: Support MoH in the development of clinical protocols on “patient transportation in perinatology”; qualification standards for neonatologists; new teaching materials including eLearning materials; regulatory support and national dissemination of the developed “products”.

Establishment of Oblast Management Teams as program leaders and coordinators in the regions; thematic officers (training, monitoring, ICT); experts responsible for steering and managing all program activities at the oblast/rayon level, resource planning and mobilization, networking and communication.

## Appendix C. Ukrainian–Swiss Project “Medical Education Development” (2018–2023)

The ‘Strategy for the Development of Medical Education in Ukraine’ was published by Ministry of Health of Ukraine (MoH) in July 2018. This strategy suggested changes on all levels of medical education, including undergraduate, postgraduate, and continuing professional development (CPD). An important aim was to enhance the enrolment requirements for students wishing to enter the medical faculty. To this end, the internationally recognized exam from the International Foundations of Medicine (IFOM) was to be introduced. Further, orientation towards higher scientific and clinical skills standards as well as implementation of the principles of professional integrity was aimed at the juxtaposition of Ukrainian medical education and that of the European Union. Due to the COVID-19 pandemic and the full-scale invasion by Russia, these priorities and objectives have mostly not been achieved. Nonetheless, new CPD policies have improved training opportunities for medical doctors, and are expected to do the same for nurses as of 2023.

Goal of the first phase of the project (December 2018–June 2023): the availability of adequately qualified, trained, and motivated workers in the health sector are critical factors for delivering quality healthcare interventions to improve population health outcomes. Thus, the SDC and MoH recognize the importance of fostering the systematic development of human resources, with a focus on frontline healthcare workers and healthcare managers.

Embedded into the Swiss cooperation program strategy with Ukraine, the overall goal of the Medical Education Development Project (MED) is to provide quality care to the Ukrainian population by improving the medical education system for primary healthcare workforce. Specific objectives include:

- Strengthening the capacities and skills of primary healthcare staff (family doctors and nurses) through an improved national medical education system, and
- Improving the performance and efficiency of healthcare services through enhanced management capacities.

### Appendix C.1. Thematic Project Areas

A. Primary healthcare sector. The project contributes to a new Primary Health Care model. This includes newly defined roles for nurses, who work in teams with General Practitioners. A proven format of professional enhancement is the organization of expert communities, so-called ‘peer groups’, that aim at the development of skills and solve problems in a self-managed format.

The MoH announced that peer groups are recognized as a formal CPD activity, ensuring CPD points for healthcare providers.

B. Medical educational policies and practices. Medical education reform requires structural changes. New regulatory documents are elaborated to enable the introduction of new policies, especially related to postgraduate education and continuing professional development.

### Appendix C.2. Achievements

The project has systematically strengthened six partner education institutions (see Beneficiaries) across the country to assist in developing their autonomous status.

- Improvement of clinical skills: Simulation based training centres had previously been build up. Students and professionals now benefit from practice-oriented, well-equipped, and organised clinical skills laboratories. Exercises in real-life clinical scenarios lead to higher competencies and fuel motivation.
- Educational competencies for educators are substantially enhanced in terms of new knowledge and didactic methods and to implement a student-oriented approach.
- Research capabilities: medical training institutions were traditionally hardly engaged in conducting research. A research capacity-building activity was launched in 2021 to train research techniques and to conduct of concrete research projects.
- Multiple guides, courses, and encounters with practitioners and educators have gradually changed the educational landscape. Focus topics include family medicine, mentorship for healthcare educators, simulation approaches in medical education, digital learning platforms, and ‘competence-based education in healthcare’.
- Empathy and humanity in healthcare: a guide and course was developed by medical educators and facilitated by a patient’s rights advocate, leading to better interaction with patients.

Regional exchanges. Primary care reform along with medical education reform is highly relevant for all countries in transition. Therefore, the project actively supports policy and practice exchanges and partnerships with countries that have already implemented similar changes, such as Poland, Baltics, Tajikistan, and Croatia.

Healthcare management. Better governance in healthcare is needed for reforms. Therefore, a new Master’s program on healthcare management was developed, along with online courses. Key topics include healthcare system financing, quality management, strategic management for healthcare facilities, fundraising for healthcare facilities, and financial management. More than 2000 participants have enrolled in online courses.

High-level events for healthcare leaders (educators, managers, medical doctors, nurses, researchers, NGOs, etc.) were staged jointly with the National Health Service of Ukraine (NHSU) and the Ukrainian Health Care Center (UHC).

## Appendix D. Guide for the Interview with Key Stakeholders

What are the priorities for the development of mother and child health in Ukraine?

What activities do you think the Program adequately supported or implemented?

Do you think that the Program helps to introduce changes, and do you consider these changes to be timely, necessary, and effective (addressing existing issues and having expected results)? How do you measure/define this?

What strategies, approaches, and actions made these changes possible?

Were there any negative consequences of the Program activities? What strategies, approaches, and actions led to such consequences?

Were you able to establish new partnerships or join a new professional network (internal or external) to promote changes in mother and child health and collaboration?

Can you identify any achievements or changes that have occurred in the healthcare facility due to the Program activities? Please give an example./Has anything changed in your professional activities/patient treatment practices due to the Program activities? Please give an example./Do the Program activities and the chosen approach to the implementation of activities lead to changes in your healthcare facility/practice/ area compared to the previous situation? (select more relevant to the profile of the respondent)

Are you satisfied with the process of planning, needs assessment, and decision-making in the Program: is the cooperation transparent? Please provide an example.

How have you been involved in the program educational activities? How would you describe these activities?

Do you regularly communicate with the Program management/representatives? How do you communicate?

In your opinion, will the changes that the Program is introducing now be sustainable after it is approved by the facility/Ministry? Why do you think so? How will you be able to support further development?

Were there any other unplanned results achieved through the Program?

## Appendix E. Materials Developed by the Swiss–Ukrainian Program in Mother-and-Child Health (2008–2015)

### Resources for Healthcare Professionals

(1) Manuals

- Stabilization of newborns before transportation and support during transportation
- Respiratory support of newborns
- Pregnancy and childbirth in women with scar on uterus after Cesarean section
- Multiple Pregnancy manual
- Effective antenatal care manual
- Physiological pregnancy care manual for trainers

Clinical manuals describe relevant issues of obstetrics and neonatology. They consist of discrete and comprehensive thematic modules that allow the teaching of each module individually or in appropriate combinations with other modules (depending on the needs of learners). Each manual consists of two parts: a guide for learner and a guide for trainer. All manuals are based on the best international practices and approaches; however, they are most adapted to Ukrainian realities. Manuals are available in Ukrainian and Russian. They are officially approved by the National Medical Academy of Postgraduate Education and the Ministry of Health and recommended for use throughout the territory of Ukraine.

The manual is based on the latest international evidence on clinical practice for general practitioners. It is aimed to help family doctors in performing diagnosis and providing pregnancy care. The manual updates doctors’ knowledge in detecting threatening conditions during pregnancy and, if necessary, to provide emergency care. This manual is a guide for trainers of post-graduate courses. Its curriculum and standardized teaching program are approved by the Ministry of Health of Ukraine.

- Guide on Telemedicine in clinical practice

This manual describes the main aspects of telemedicine implementation and functional principles of the telemedicine platform ‘iPath’ introduced by the program. It consists of two parts-one for learner and other for trainer.

(2) e-Learning Modules

- Natural childbirth
- Multiple pregnancy
- Continuous positive airway pressure as method of newborn respiratory support
- Primary resuscitation of newborns
- Shoulder dystocia in newborns

e-Learning modules constitute an important program innovation. For the first time, written information is combined with audio, photo and video materials. These electronic materials are included into the NMAPE series of post-graduate courses and recommended for use by the Central methodical cabinet of the high medical education of MoH of Ukraine. The advantage of these manuals is the possibility to use them both for self-education and for training in groups.

- Manual “Electronic Training Manuals for Reflection of Medical Procedural Knowledge: principles, stages of elaboration, and methodology”

This manual was developed by NMAPE and recommended by the Ministry of Health of Ukraine for lecturers of higher medical institutions. It presents the experience of developing electronic manuals within the mother-and-child health program.

(3) Informational Materials

- Practical aspects of neonatal care handbook

This handbook is a concise and easily readable clinical decision guide that contains many tables and diagrams, useful for everyday medical practice, namely: tables for calculating the doses of medicines and antibiotics, an indicative panel of respiratory disorders and action algorithms, such as for sepsis, etc.

- Wall posters for obstetric departments and NICUs

Posters contain useful information for daily medical practice, namely: diagrams, tables, and algorithms. They are made in a convenient format that allows you to hang them on the wall and always have the necessary information before your eyes.

- Software to calculate nutrition for patients in neonatal intensive care units

This easy-to-use software tool developed in a spreadsheet format (Excel) can generate a prescription chart for a newborn that requires intensive care, enteral and parenteral nutrition.

- Manual for NICUs nurses

The manual describes core interventions and steps of providing care for children in neonatal units. The publication pays much attention to creating a safe environment for patients, the optimal sequence of actions when performing clinical interventions. Further, communication skills with parents of a sick child are elaborated. All information presented in separate chapters that contain clear recommendations, photos and illustrations.

- Guide on monitoring visits

Approaches and tools for conducting monitoring visits in a medical facility are outlined. The guide is complemented by monitoring forms used to collect information during the visit. The document is available in Ukrainian and Russian.

Materials for Healthcare Managers

- Manual “How to improve perinatal care in Ukraine?”

This publication outlines the essentials of the program implementation experience during 2008–2010. The results of the program activity are introduced in the form of four models. Each model highlights specific aspects of perinatal care provision and offers innovative approaches to improve clinical and managerial practice. All four models form integral part of a systems building approach. The program’s experience proves that perinatal practices’ improvement is possible only due to complex implementation of approaches and tools described in the publication

- Health technology management manual

The manual is devoted to the proper management of modern medical equipment with a special emphasis on the elaboration of an electronic inventory. It consists of two parts (for learners and trainers) and can be used for training.

- Informational package “Swiss-Ukrainian Mother and Child Health Program. New approaches to healthcare organization. Achievements. Implementation experience. Recommendations”
- Handbook “Establishing a Simulation Center: Essentials and Guidelines”

This package consists of a booklet and a DVD that contain information about the program main products and achievements as well as practical recommendations on their implementation in other regions of Ukraine. Also, all program products and materials are recorded on DVD.

The handbook is divided in two main parts: the first part describes the concepts related to the establishment of Medical Simulation Centers, the role of simulation-based training in medical education (SBME). It reviews national and international experiences of establishing of these types of institutions. The second part provides the methodology and tools to develop and implement mother and child health Simulation Centers, including topics such as organizational capacity building, technology requirements, curriculum development and economic considerations. A variety of experts with experience in implementing SBME have contributed to this handbook. The handbook is available in English and Ukrainian.

Regulatory Documents

- Perinatal Service Package

This document outlines a structured description of health services which are essential, effective and accessible to the entire population according to levels of care. The ‘Package’ helps planning, controlling, and analyzing the provision of integrated perinatal care services and, therefore, improves the results. It is authorized by the Ministry of Health of Ukraine–Order № 204 of 13.04.2011.

- Guideline on transportation of high perinatal risk newborns in Ukraine (approved by the Ministry of Health of Ukraine, Order №1024 of 28.11.2013)
- Clinical guideline on primary, resuscitation and post-resuscitation care to newborns in Ukraine (approved by the Ministry of Health of Ukraine, Order №225 of 28.03.2014p.)
- Guideline on the transportation of pregnant women, woman in childbirth and women recently confined in terms of regionalization of perinatal care in Ukraine (approved by the Ministry of Health of Ukraine, Order №51 of 06.02.2015p.)
- Guideline on providing medical care for women with multiple pregnancy (approved by the Ministry of Health of Ukraine, Order №205 of 08.04.2015p.)

All guidelines are optimized to Ukrainian healthcare system. They are based on the latest findings in medicine, contain key principles of a specified subject area and aimed to help medical personnel in the improvement of clinical practices and achieving the most positive outcome for the patient.

- Qualification standards for neonatologists

This document describes knowledge and skills that should be mastered by neonatologist for providing qualitative care for patients. It is complemented by descriptions of tasks and duties. It is developed for the needs of the medical education system in Ukraine.

Informational Materials for Parents

- Informational brochure for pregnant women: “My Pregnancy Diary”

This publication aims at helping pregnant women and their families in preparation for childbirth and child care during the first months after birth. The content of the “Diary” corresponds to best practice approaches in safe motherhood. It is recommended for publishing and the use within facilities of the Ministry of Health of Ukraine.

- Diary for parents with children admitted in intensive care

The publication aims at helping parents to understand better the specifics of care after a child was taken to the neonatal intensive care units (NICUs) and to facilitate communication between medical professionals and parents. It is written in a simple way and includes answers to many questions frequently asked by parents. The Diary is available in Ukrainian and Russian version. It was disseminated among maternity houses and children’s hospitals in Ukraine.

Printed versions of most materials were disseminated among all regions of Ukraine (regional healthcare departments, hospitals, medical libraries and medical universities). Electronic versions are available on the program website (http://motherandchild.org.ua/, accessed on 25 June 2023) in the section “Resources”. All program publications are distributed on the cost-free bases. Commercial use (sale) is forbidden.

## Appendix F. Stakeholders’ Feedback on Swiss–Ukrainian Mother-and-Child Collaboration

When we talk about the healthcare reform, we understand that everything so far is called ‘reform’ however it has cosmetic nature, i.e., nails are cut and the rest remained the same. In order to bear radical reforms, there is need as political will as readiness of society to these reforms as discussions as funding as well as stable situation in the country. Respectively, we cannot be successful in healthcare reform without changing the system of training doctors and current postgraduate education, therefore, these processes should go in parallel. The Program is a catalyst for these changes (professor, medical doctor).

When we started in 2004 to conduct training, we heard “We will be taught? It is funny, because we have 300 years of experience, we know how everything happens”. When these training were finally conducted-and we spoke there about things that they believed were correct for 300 years, and it turned out that it is a myth that has no ground under,–many doctors apologized for their hostile perception in the beginning (medical doctor-trainer).

Thanks to the Swiss program we learned much more than we were taught in institutes. We took part in clinical training, but this case [Nastia, newborn with extremely low birth weight] was our first independent practice and success in nursing children with such critical weight (neonatologist, Volyn region).

The feedback of neonatal transport team could be treated as the positive outcome criterion. When I ask if there any positive changes in neonatologists’ activity, they reply “yes, when we come, as a rule, the child is stabilized and we could take him to the regional hospital”. I ask how it was in the past and they answer “we received a call that a sick child was born but no measures were taken and when we came it took us about 3–5 or even more hours to stabilize the newborn and after that we could take him to the NICUs. I think it is the great positive outcome of coaching work” (regional chief neonatologist).

Owing to the program’s cooperation we had the opportunity to implement the state-of-the-art perinatal technologies in the hospitals. The readiness to provide urgent assistance was significantly improved, the local clinical protocols were developed, the medical staff knows how to behave in some difficult situation. It was reflected on clinical indicators. After the tactic of labor process was changed (according to evidence-based medicine guidelines), the number of normal births has increased from 28% in 2007 to 82%; the number of labor anomalies and birth complications has decreased in three times, the number of labor bleedings has reduced from 10,3% to 2,8%. The perinatal losses indicator is lower than the average in the region. About 65% cesarean sections are performed under the spinal anesthesia and it was not practices till 2008. After the program’s training the vaginal birth after cesarean section has been introduced into the practice and in 2013 the percentage of them was 25%. (Hospital Deputy Head)
